# The Effect of Photobiomodulation Therapy in the Treatment of Postoperative Pain After Impacted Third Lower Molar Extraction: A Systematic Review with Meta-Analysis

**DOI:** 10.3390/dj14090588

**Published:** 2026-09-11

**Authors:** Leopoldo Mauriello, Vitolante Pezzella, Andrea Blasi, Giuseppe Trapanese, Elio Ramaglia, Vincenzo Iorio-Siciliano, Luca Ramaglia

**Affiliations:** 1 Department of Neuroscience, Reproductive Science and Dentistry, University of Naples Federico II, 80131 Naples, Italy; leopoldo.mauriello@unina.it (L.M.); vitolante.pezzella@unina.it (V.P.); giuse.trapanese@studenti.unina.it (G.T.); vincenzo.ioriosiciliano2@unina.it (V.I.-S.); 2 Department of Clinical Medicine and Surgery, University of Naples Federico II, 80131 Naples, Italy; elio.ramaglia@unina.it

**Keywords:** photobiomodulation, impacted third molar, postoperative pain

## Abstract

**Background/Objectives:** Postoperative pain following impacted mandibular third molar extraction is a common complication that negatively affects patients’ quality of life. Photobiomodulation (PBM) therapy has been proposed as a non-pharmacological adjunctive approach to reduce postoperative pain. This study aimed to evaluate the effectiveness of intraoral PBM in reducing postoperative pain after impacted mandibular third molar extraction compared with placebo, sham treatment, or standard postoperative care. **Methods:** A systematic review was conducted according to the PRISMA 2020 guidelines. PubMed and Embase were searched through January 2025 for randomized controlled trials (RCTs) investigating intraoral PBM after impacted mandibular third molar extraction. Only studies reporting postoperative pain using the Visual Analog Scale (VAS) were included. Risk of bias was assessed using the Cochrane RoB 2 tool, and the certainty of evidence was evaluated using the GRADE approach. Random-effects meta-analyses were performed at 24, 48, and 72 h postoperatively using standardized mean differences (SMDs). **Results:** Eight RCTs met the inclusion criteria for qualitative synthesis, whereas only two provided extractable data for quantitative analysis. Meta-analysis demonstrated a significant reduction in postoperative pain in the PBM group at 24 h (SMD = −0.64; 95% CI: −0.90 to −0.38; *p* < 0.001), 48 h (SMD = −0.82; 95% CI: −1.64 to −0.01; *p* = 0.047), and 72 h (SMD = −1.11; 95% CI: −1.38 to −0.84; *p* < 0.001). **Conclusions:** Intraoral PBM appears to reduce postoperative pain during the first 72 h. However, the available evidence is limited by the small number of studies included in the meta-analysis, heterogeneity in PBM protocols, and inconsistent outcome reporting. Additional well-designed RCTs are needed to strengthen the current evidence.

## 1. Introduction

After oral surgery, one of the most common sequelae is pain, which represents a complex neurobiological phenomenon due to the interaction between peripheral tissue damage and central response. The surgical trauma always releases proinflammatory cytokines (e.g., IL-1, IL-2, and TNFα), prostaglandins, and bradykinin that work to reduce nociceptor thresholds and increase neural transmission [1]. The increase in pain sensation is also due to the transient activation of glutamatergic pathways at specific receptors, together with mitochondrial metabolic alterations; these interactions further increase hyperalgesia and allodynia [1]. Several experimental models have demonstrated that the reduction or even modulation of these phenomena can reduce nociception, further supporting the development of non-pharmacological strategies for pain control [1]. The extraction of an impacted third lower molar is one of the most common procedures in oral surgery and represents a source of worry for patients in terms of postoperative pain [2,3]. Even though it is considered a routine procedure in daily practice, surgery is often associated with pain, edema, and trismus in the early postoperative phase [3]. After 24–48 h, the highest pain intensity is generally recorded, then it decreases in the following days; however, it may interfere with nutrition, speech, sleep, and overall quality of life [3]. Bone removal, surgical difficulties, duration of the procedure, and individual response may be good predictive factors for pain development [2]. Conventional treatment relies on the use of non-steroidal anti-inflammatory drugs, corticosteroids, and, in rare cases, opioids, but all these medications are widely associated with collateral effects (e.g., gastrointestinal, renal, and systemic adverse effects [3,4]), so, despite their reliability, not all patients can safely take them [5]. Therefore, there is increasing interest in the development of adjunctive methods to reduce pain and avoid systemic collateral effects. Photobiomodulation (PBM) therapy is one of the non-pharmacological modalities with both analgesic and anti-inflammatory properties. PBM employs red or infrared light to promote photochemical effects without any thermal damage. The main target is the mitochondrial C-oxidase since its activation is capable of enhancing adenosine triphosphate (ATP) synthesis, regulates reactive oxygen species, and modulates intracellular calcium signaling [6]. The PBM can therefore down-regulate inflammatory mediators and improve microcirculation, thus providing a valid rationale for pain modulation [7,8]. Light irradiation seems to reduce the nociceptive responses of capsaicin, formalin and glutamate and may act on both central and peripheral components [9]. Additionally, acceleration of tissue repair was recorded after PBM exposure, which seems to modulate cytokine expression [10,11]. These effects are particularly relevant in third lower molar surgery, where bone removal and soft-tissue manipulation are common inducers of both inflammatory response and manipulation of nociceptors [12]. Thus, the direct intraoral delivery of light allows irradiation of both the post-extraction socket and surrounding tissues, counteracting the inflammatory cascade [13]. Several studies have recently explored the potential of PBM after third molar extraction using different wavelengths and energy densities, as well as different application methods [14,15,16]. However, the heterogeneity of protocols and outcomes has limited the comparability of results as well as the proposition of a unique protocol. There are several differences that were recorded according to irradiation session, intra- vs. extraoral approach, total energy delivered, and wavelength [5]. Furthermore, pain evaluation has been variable among studies, although the most reliable and commonly used pain assessment was the Visual Analog Scale (VAS) [17]. Generally, the first 72 h are particularly relevant in terms of pain development and resolution since they correspond to the peak of the inflammatory response [18]. Thus, evaluating the possible effect of PBM could provide clinical improvement in the reduction in VAS scores and therefore a reduction in the use of painkillers, causing less stress on the body and preventing unwanted collateral effects. Despite the increasing use of PBM in oral surgery, the available evidence remains difficult to interpret because of substantial differences among treatment protocols and variability in reported clinical outcomes. Differences in wavelength, energy parameters, irradiation sites, number of sessions, and intraoral or extraoral application may influence the biological and clinical response, limiting direct comparison among studies. Moreover, although several randomized trials have investigated the effect of PBM on postoperative pain after mandibular third molar extraction, the magnitude and consistency of the analgesic effect remain uncertain. A focused synthesis of the available randomized evidence is therefore needed to clarify the effectiveness of intraoral PBM and to identify the main methodological and treatment-related factors that may contribute to differences in clinical outcomes. Therefore, the present systematic review and meta-analysis was designed to evaluate whether intraoral PBM therapy, compared with standard care or placebo, could represent a valid alternative in reducing postoperative pain compared with standard treatment. The main outcome was the analysis of Visual Analog Scale scores within the first 72 h after surgical extraction of the mandibular third molar, with the goal of providing clinical guidelines and identifying a common protocol.

## 2. Materials and Methods

### 2.1. Protocol and Registration

The systematic review was conducted according to the PRISMA statement 2020. The protocol was registered on the PROSPERO database (registration number: CRD420261285303). All procedures were decided “a priori,” including inclusion and exclusion criteria, outcomes, and statistical analysis (if feasible).

### 2.2. Eligibility Criteria (PICO Framework)

Population (P):

Patients who underwent surgical extraction of an impacted third lower molar.

Intervention (I):

Intraoral application of laser or PBM therapy in the peri- or postoperative period following extraction, regardless of wavelength or laser type (e.g., diode, Er:YAG, Nd:YAG, CO_2_).

Comparator (C):

Standard postoperative care, placebo/sham laser, or no treatment.

Outcome (O):

Postoperative pain measured exclusively using the Visual Analog Scale (VAS) within the first 7 days after surgery. Studies using alternative pain scales were not included.

### 2.3. Study Design

Only randomized controlled trials (RCTs) published as full texts were included. Multi-arm trials were eligible if at least one comparison matched the PICO; only intraoral-laser-versus-control data were extracted for synthesis.

### 2.4. Inclusion Criteria

- Human patients undergoing surgical extraction of the impacted third lower molar.
- Intraoral laser application in the peri- or postoperative period. Any laser type or wavelength.
- Quantitative assessment of postoperative pain using the Visual Analog Scale (VAS) only.
- Pain assessed at least once within 7 days post-surgery.
- Randomized controlled trials (RCTs).
- Full-text original articles in peer-reviewed journals.
- English (or other languages that can be critically appraised).
- Multi-arm studies: Included if at least one arm matches the PICO; only intraoral-laser-vs.-control comparisons will be used in quantitative synthesis.

### 2.5. Exclusion Criteria

- Animal or in vitro studies.
- Studies not involving mandibular third molar extraction (if data are not separable).
- Interventions with exclusively extraoral laser application; laser used only as a surgical tool (e.g., incision/osteotomy) without peri- or postoperative application; or non-separable combinations (laser plus another experimental treatment).
- Use of pain scales other than VAS (e.g., NRS, Likert, verbal scales).
- Absence of a quantitative pain outcome.
- Case reports, case series, observational studies without a control group, reviews, editorials, and letters.
- Abstracts without available full text.
- Incomplete or non-extractable data.
- Duplicates or multiple reports of the same sample.

### 2.6. Information Sources and Search Strategy

A systematic search was performed in PubMed and Embase without time limits up to the last search date (18 January 2025). Full electronic strategies were developed according to the following search strings:

- PubMed:

((“third” OR “thirds”) AND (“lower” OR “lowered” OR “lowering” OR “lowers”) AND “molar” AND (“post operative” AND “pain”) AND (“photobiomodulation” OR “low level light therapy”)) AND randomizedcontrolledtrial[Filter]

- Embase:

(‘third lower molar’ OR (third AND lower AND molar)) AND (‘post operative pain’) AND (‘low level laser therapy’) AND ‘randomized controlled trial’/de

Search strategies were developed separately for each database according to its specific indexing system. Therefore, search strategies were adapted rather than identical (MeSH and Emtree, respectively), while maintaining the same conceptual domains related to mandibular third molar extraction, postoperative pain, and photobiomodulation/low-level laser therapy. The complete search strategies for PubMed and Embase are provided in Supplementary Table S1.

### 2.7. Study Selection Process

Records retrieved from the databases were imported. Study selection was performed independently by two reviewers (LM and LR). Titles and abstracts were initially screened according to predefined eligibility criteria, followed by full-text assessment of potentially eligible studies. Disagreements regarding study eligibility were resolved through discussion until consensus was reached (Supplementary Table S2).

### 2.8. Data Extraction

Data extraction was performed independently by two reviewers (LM and LR) using a standardized data extraction approach. The following information was collected:

- Study characteristics;
- Sample size and demographics;
- PBM protocol parameters (including wavelength, laser type, output power, energy, energy density, irradiation area, irradiation time, application sites, and treatment schedule);
- Postoperative pain outcomes: VAS pain values at 24, 48, and 72 h (mean ± SD).

Any disagreement during data extraction was resolved through discussion and consensus. When multiple time points were available, only data within 72 h were considered. If more than one intervention arm was present, only comparisons matching the PICO were extracted. When relevant information was not available in the original publications, data were reported as NR (not reported). No assumptions or indirect calculations were performed to estimate missing PBM parameters. Studies with unavailable quantitative data were retained for qualitative synthesis but were not included in the meta-analysis.

### 2.9. Statistical Analysis

Quantitative synthesis was restricted to trials reporting mean ± SD for VAS pain. Three meta-analyses were conducted at 24 h, 48 h, and 72 h, to avoid unit-of-analysis errors due to correlated repeated measures. Effect size was expressed as standardized mean difference (Cohen’s d) using a random-effects model implemented in SPSS v.30. Both studies included in the quantitative synthesis used a split-mouth design. Although paired analyses accounting for within-subject correlation are preferable for this design, the original publications did not provide the parameters required to calculate paired effect estimates, such as within-subject standard deviations of the differences or correlation coefficients between intervention and control sites. Therefore, the meta-analysis was performed using the available summary statistics reported in the original studies (mean and standard deviation). This limitation was considered when interpreting the pooled estimates. Heterogeneity was assessed through I^2^ and the Q test. Forest plots were generated for each time point. Although the two included studies for quantitative synthesis assessed pain using the same 0–10 Visual Analog Scale, standardized mean difference (Cohen’s d) was selected because of the relevant clinical and methodological heterogeneity between trials, including differences in PBM wavelength, irradiation protocols, energy parameters, treatment schedules, and reported outcome variability. This approach allowed standardization of the magnitude of the intervention effect across studies.

### 2.10. Risk-of-Bias Assessment

Methodological quality was evaluated using the Cochrane RoB 2 tool across five domains:

1. Randomization process;
2. Deviations from intended interventions;
3. Missing outcome data;
4. Measurement of the outcome;
5. Selection of the reported result.

Two reviewers evaluated each domain independently as low-risk, some concerns, or high-risk. Disagreements were resolved by discussion.

### 2.11. Certainty of Evidence (GRADE)

The overall certainty of the evidence for each time point (24, 48, 72 h) was evaluated using the GRADE approach considering: risk of bias, inconsistency, indirectness, imprecision, and publication bias. Evidence from RCTs started as high and was downgraded when serious limitations were identified. A Summary-of-Findings table was planned.

## 3. Results

### 3.1. Study Selection

**Figure 1:**
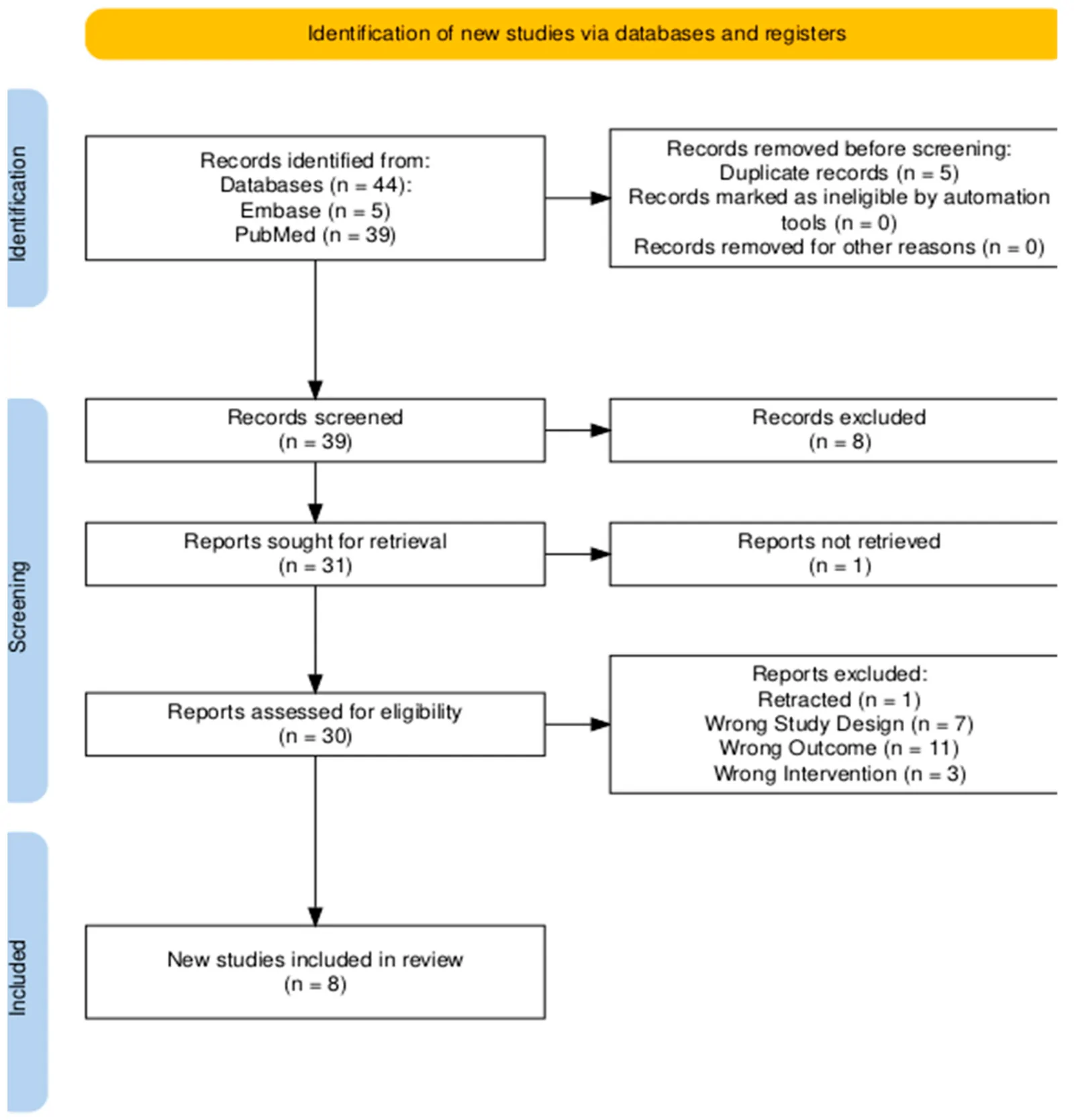
PRISMA 2020 flow diagram of study selection.

The selection process followed the PRISMA 2020 framework (PRISMA flow diagram Figure 1). A total of 44 records were identified (PubMed n = 5; Embase n = 39). After removal of 5 duplicates, 39 records were screened. Eight records were excluded at the title/abstract screening stage, and one additional record could not be retrieved. Therefore, 30 full-text articles were assessed for eligibility. Eventually, only eight studies were included in the review, of which only two presented an extractable mean and standard deviation at the predefined points (24, 48, 72 h) and were therefore used for meta-analysis.

### 3.2. Characteristics of Included Studies

The characteristics of the eight included trials are summarized in Table 1, while the characteristics of photobiomodulation protocols of the included studies are summarized in Table 2.

The evaluated studies analyzed patients who underwent surgical extraction of impacted third lower molars, treated with intraoral PBM alone or with extraoral irradiation; studies that applied only extraoral irradiation were not included. The studies were compared with sham, placebo, or standard postoperative care. The samples ranged from 9 to 83 patients. Most of the included studies were conducted according to a split-mouth randomized design, while the diode laser was the most frequently used, with variable wavelengths (from 670 to 980 nm) and heterogeneous irradiation protocols. Postoperative pain was evaluated exclusively with VAS, varying from the first to the seventh day. Six randomized trials were included only in the qualitative synthesis because data pooling was not possible due to either non-extractable intrametric data or incompatible reporting formats. Momeni et al. [16] presented postoperative pain using non-parametric statistics and graphical representations without reporting mean ± SD values at 24–48–72 h, thus preventing the calculation of standardized effect sizes. Sekerci et al. and Uzeda et al. [6,19] investigated combined intra- and extraoral PBM protocols but reported VAS outcomes as medians or cumulative scores, with no separable group-level descriptive statistics. Neelofar et al. [20] provided pain trajectories over seven days yet expressed results through ordinal categories rather than continuous means. Farhadi et al. and Ferrante et al. [15,21] had swelling and trismus as their main outcomes; although VAS was recorded, the data were not compatible with independent-group meta-analysis. Despite this, the included studies showed a heterogeneous clinical trend, since some reported a reduction during the first few postoperative days while others found no statistically significant difference with control groups. Detailed characteristics of the photobiomodulation protocols applied in the included studies are reported in Table 2. The included trials showed substantial variability in laser parameters, including wavelength, power output, energy delivery, irradiation time, application sites, and treatment schedules. When specific parameters were not reported or were unclear in the original publications, they were reported as NR (not reported).

### 3.3. Risk of Bias

**Figure 2:**
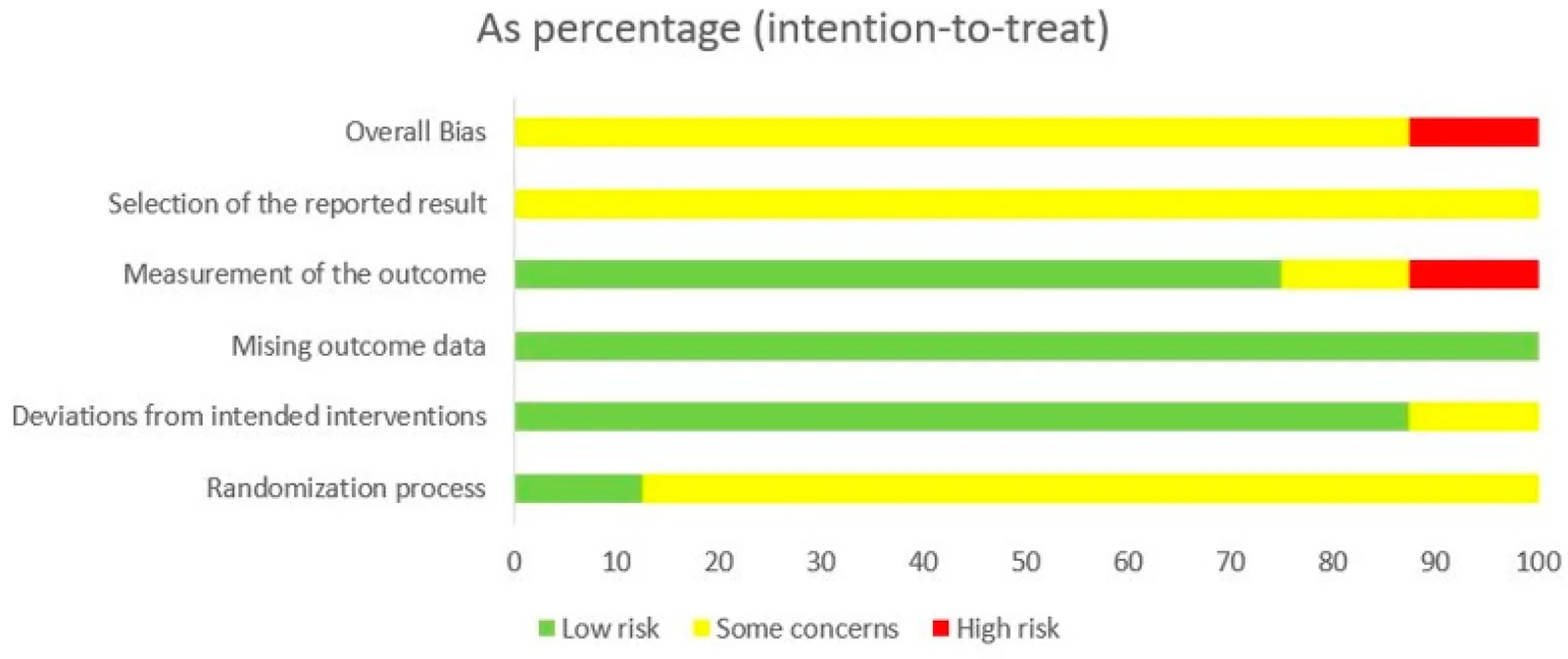
Risk-of-bias summary graph.

**Figure 3:**
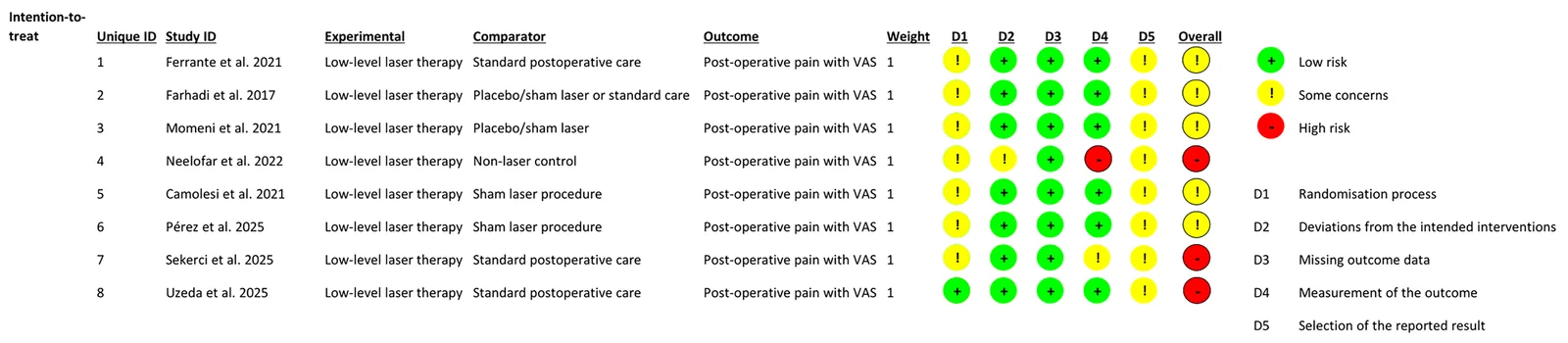
Risk-of-bias traffic-light plot (RoB 2) [6,12,15,16,17,19,20,21].

Assessment using the RoB 2 tool revealed overall acceptable methodological quality. Two trials were judged at low risk of bias, four presented some concerns mainly related to the randomization process and deviations from intended interventions, and two were rated at high risk due to incomplete reporting of allocation concealment and outcome assessment. The detailed summary graph and traffic-light plot are presented in Figure 2 and Figure 3.

### 3.4. Quantitative Synthesis

**Pain at 24 h**

**Figure 4:**
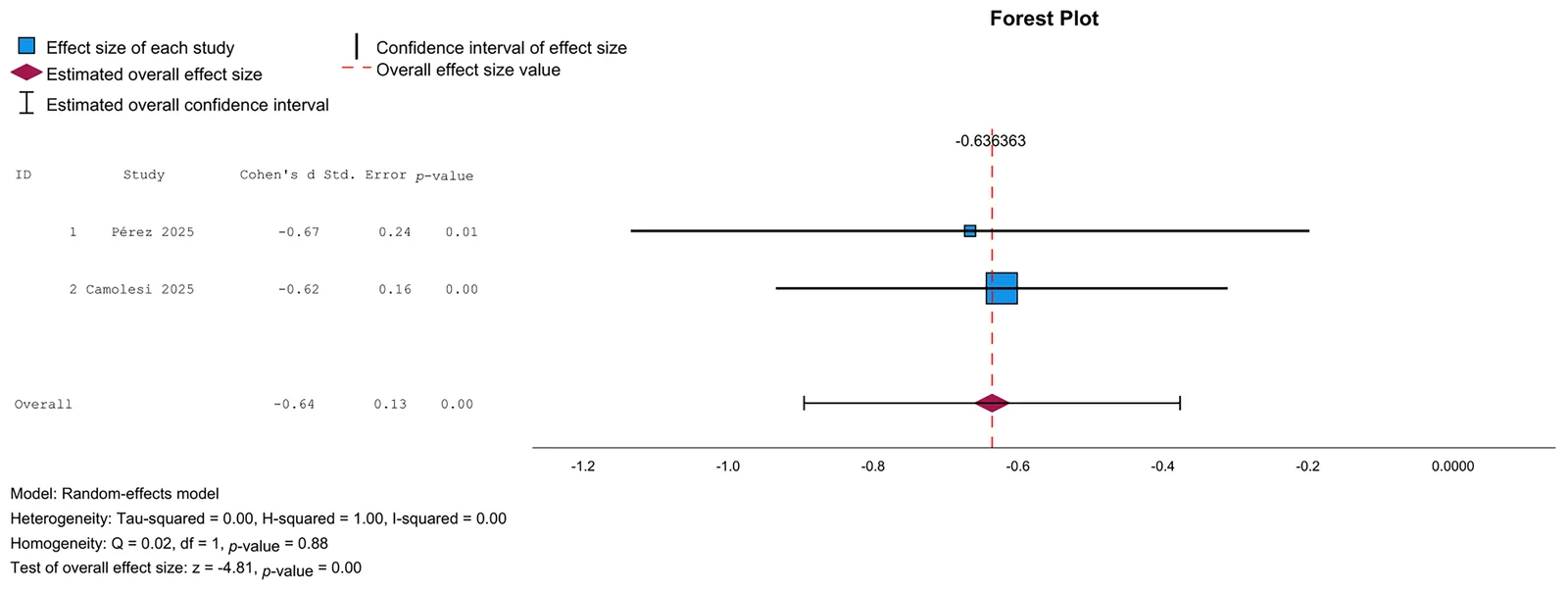
Forest plot of VAS at 24 h [12,17].

Pérez 2025 and Camolesi 2025 [12,17] found a statistically significant reduction in postoperative pain at 24 h in the PBM group (Cohen’s d = −0.64; 95% CI: −0.90 to −0.38; *p* < 0.001). Heterogeneity was negligible (I^2^ = 0%, Q = 0.02, *p* = 0.88). The forest plot is shown in Figure 4.

**Pain at 48 h**

**Figure 5:**
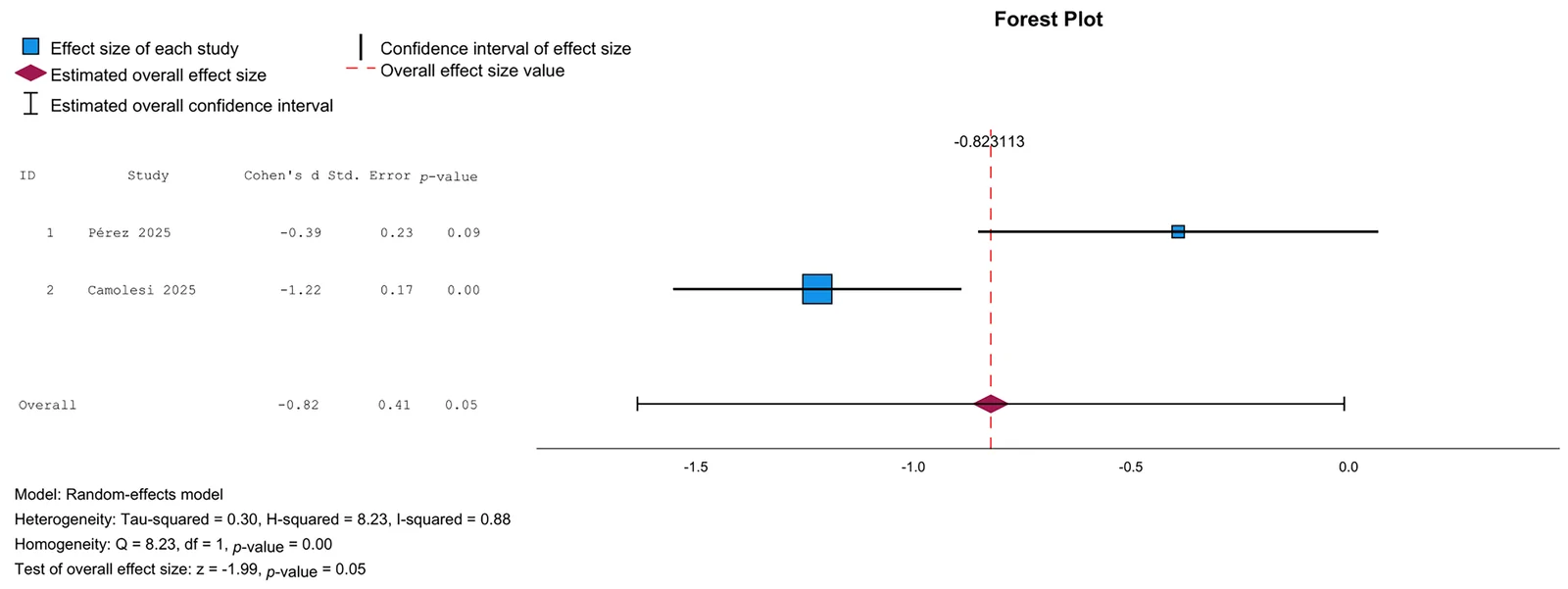
Forest plot of VAS at 48 h [12,17].

At 48 h, the pooled effect was still statistically significant (d = −0.82; 95% CI: −1.64 to −0.01; *p* = 0.047) but with substantial heterogeneity (I^2^ = 88%, Q = 8.23, *p* = 0.004). Results are displayed in Figure 5.

**Figure 6:**
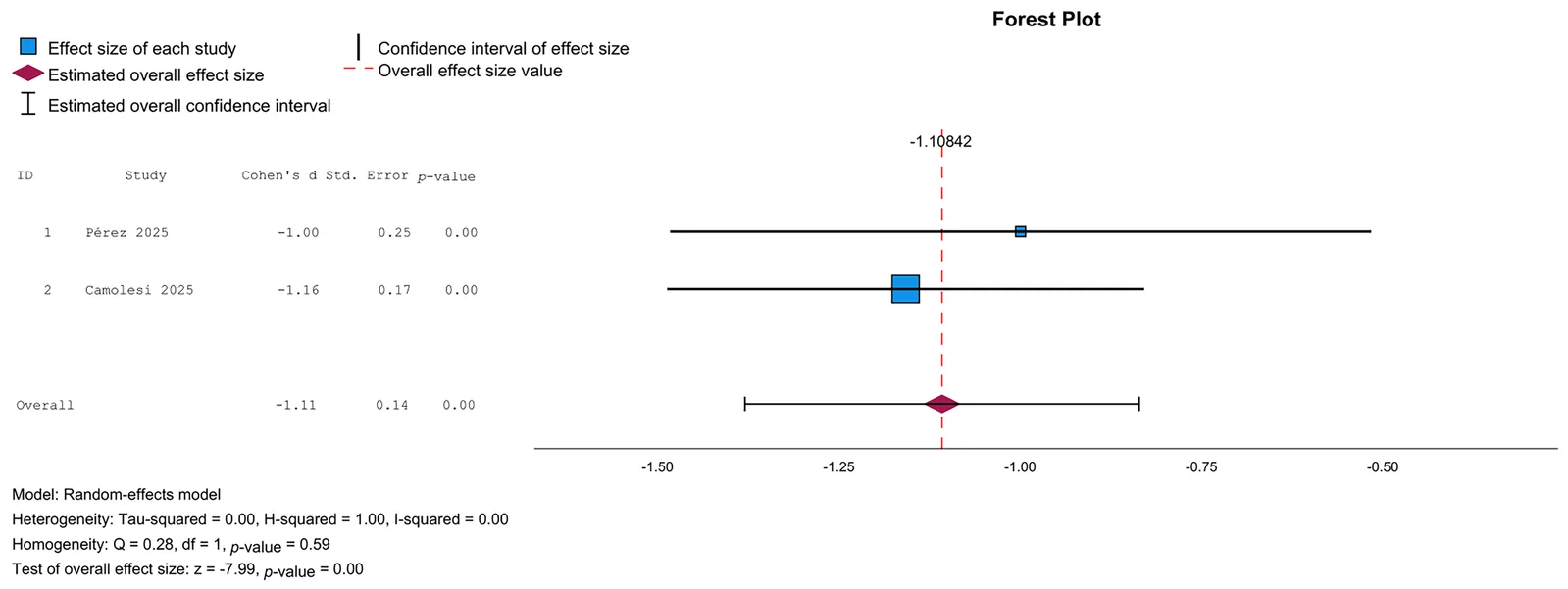
Forest plot of VAS at 72 h [12,17].

At 72 h, the pooled effect showed a statistically significant reduction in postoperative pain in favor of the PBM group (Cohen’s d = −1.11; 95% CI: −1.38 to −0.84; *p* < 0.001). Heterogeneity was negligible (I^2^ = 0%, Q = 0.28, df = 1, *p* = 0.59). The forest plot is shown in Figure 6.

However, all these pooled estimates were based on only two studies and therefore should be interpreted considering the limited quantitative evidence available.

### 3.5. Certainty of Evidence

According to the GRADE approach, the certainty of evidence was rated as moderate at 24 h and 72 h and low at 48 h due to inconsistency and imprecision. The Summary of Findings is reported in Table 3.

### 3.6. Explanations and Downgrading Rationale

- Risk of bias: Not downgraded—the two trials included in the meta-analysis showed low risk of bias according to RoB 2.
- Inconsistency: Downgraded one level at 48 h due to substantial heterogeneity (I^2^ = 88%).
- Indirectness: Not downgraded—population, intervention, comparator, and outcome fully matched the PICO.
- Imprecision: Downgraded one level at 48 h because of wide confidence interval and small number of studies.
- Publication bias: Not assessed due to fewer than 10 studies.

## 4. Discussion

### 4.1. Main Findings

The main aim of the present systematic review was to assess the effectiveness of intraoral PBM therapy as an adjunctive strategy to control postoperative pain after third lower molar surgical extraction. Postoperative pain in third lower molar surgery still represents a frequent and clinically relevant issue. Most of the time, the pain reaches its apex at 24–72 h due to inflammatory release after the surgical trauma [12,21]. In this phase, inadequate pain control may negatively affect the patient’s comfort, recovery, and general satisfaction [4]. From the data analyzed in the quantitative synthesis, a statistically significant reduction in pain was recorded at 24, 48, and 72 h in favor of the group treated with PBM therapy, with a moderate-to-large standardized effect size. Even though only two studies were feasible for meta-analysis, the consistency of the effect at each time point seems to suggest a reproducible analgesic effect when PBM is applied intraorally in the first postoperative phase. These results are in line with the hypothesis that PBM could be effective during the period of maximum inflammatory response after oral surgery [17]. Both studies included in the meta-analysis showed several relevant methodological features; in fact, they were conducted as split-mouth randomized clinical trials, designed specifically to reduce inter-individual variability, and the PBM protocols were implemented immediately after surgery. Pain was always assessed using the VAS, providing mean and standard deviations at specific standardized points corresponding to the peak and resolution of the inflammatory phase. However, although in both cases a consistent and statistically significant reduction in pain level was reported, supporting the reproducibility of the observed effect when intraoral irradiation and repeated applications are employed and different lasers, in terms of wavelength and power, were adopted, compromising the definition of a general protocol [12,16]. In addition, the limited number of studies and relatively small sample size indicate that the results should be evaluated with caution. The data obtained seem to be at least partially in line with the literature. Several studies consistently reported reductions in postoperative pain and painkiller use when PBM was administered after surgery, mainly when intraoral application was delivered as part of the treatment protocol [7,14,22].

### 4.2. Comparison with Previous Evidence and Clinical Implications

On the other hand, other investigations have failed to demonstrate significant benefits compared with placebo or standard care, especially when laser application was limited to extraoral sites [23,24,25], suggesting that application modality may be a crucial factor in the pain modulation effect. As previously mentioned, differences in laser parameters and irradiation protocol were the main source of heterogeneity among all the included studies. Wavelengths ranged from visible red to infrared, and notable differences were also observed in power output, energy, and density, as well as application points, frequency, and sessions. These differences together may have influenced both reproducibility and magnitude of the clinically observed effects. Since wavelength determines the optical properties of delivered light, including tissue penetration depth and interaction with target chromophores, it may affect the biological response induced by PBM. Furthermore, PBM follows a biphasic dose–response relationship, meaning that both insufficient and excessive energy delivery may reduce the expected therapeutic effect. Therefore, variations in energy density, irradiation time, and number of sessions should be considered when interpreting differences in postoperative pain outcomes among the included trials [13,23]. Finally, the current evidence does not allow identification of a superior PBM protocol, highlighting the need for standardized reporting and adequately designed comparative studies. Studies employing repeated intraoral applications tended to report more favorable pain outcomes, whereas trials using single-session or predominantly extraoral irradiation often showed weaker or non-significant effects [20]. In terms of clinical relevance, the results of this review suggest that intraoral PBM may represent an adjunctive modality for postoperative pain management following impacted mandibular third molar extraction. PBM is not invasive and is not associated with systemic effects, which makes it particularly suitable for patients with systemic pathologies, comorbidities, and pharmacological contraindications. Considering the evaluation made, it is reasonable to say that PBM should be regarded as a complementary intervention rather than a full substitute for common analgesic care. However, this interpretation should consider that the quantitative evidence was derived from only two studies, and the generalizability of the findings remains limited by heterogeneity in PBM protocols.

### 4.3. Strengths and Limitations

The main strengths of this review include the focused research question, the inclusion of randomized controlled trials only, the application of PRISMA 2020 guidelines, the assessment of risk of bias using the RoB 2 tool, and the evaluation of certainty of evidence through the GRADE approach. However, some important limitations identified in this review lie in outcome reporting and statistical methodology. Although all included studies assessed pain using the Visual Analog Scale, several trials reported results using non-parametric statistics, medians, or graphical representations without providing extractable mean ± standard deviation values at predefined time points. Therefore, these studies could only be included in the narrative synthesis but not in the quantitative analysis, despite meeting all other eligibility criteria. Several previous reviews on PBM in oral and maxillofacial surgery have reported this issue, representing a relevant obstacle to evidence synthesis and meta-analysis [17]. The RoB 2 tool was used to evaluate methodological quality, which was generally acceptable; however, some concerns were raised regarding randomization procedures and deviations from the intended intervention. The presence of incomplete reporting regarding allocation concealment further limited the reliability of some study results. Furthermore, the GRADE approach was used to assess the certainty of evidence, which was rated as moderate for pain reduction at 24 and 72 h and low at 48 h due to inconsistency and imprecision. Both studies included in the quantitative synthesis presented a split-mouth design. Although this design reduces inter-individual variability, it requires statistical approaches accounting for within-subject correlation. In the present meta-analysis, the necessary paired parameters, such as standard deviations of within-subject differences or correlation coefficients between treatment and control sites, were not available from the original reports. Therefore, the pooled estimates were calculated using the available summary statistics, and the quantitative findings should be interpreted with caution. In addition, although the two included studies for quantitative synthesis assessed pain using the same 0–10 Visual Analog Scale, standardized mean difference (Cohen’s d) was selected because of the relevant clinical and methodological heterogeneity between trials, including differences in PBM wavelength, irradiation protocols, energy parameters, treatment schedules, and reported outcome variability. Although this approach allowed standardization of the magnitude of the intervention effect across studies, mean difference may provide a more direct clinical interpretation when identical scales are used. Therefore, the pooled estimates should be interpreted considering both the standardized effect size and the limited number of available studies. Overall, this evaluation reflects the limited number of studies included in the meta-analysis and the heterogeneity observed at intermediate time points. However, it is important to note that downgrading was mainly related to reporting limitations rather than conflicting directions of effect, suggesting that future high-quality trials could strengthen the evidence base [5]. These limitations should be considered together with the impossibility of assessing publication bias due to the limited number of quantitative studies. Furthermore, long-term outcomes were insufficiently explored.

### 4.4. Future Research Directions

Future RCTs should prioritize standardized PBM protocols and uniform outcome reporting, including predefined assessment time points and consistent reporting of mean ± standard deviation values.

## 5. Conclusions

Current evidence suggests that intraoral PBM may represent a promising adjunctive intervention for reducing postoperative pain after impacted mandibular third molar extraction, particularly during the early postoperative phase (24–72 h). Although the available evidence indicates a potential analgesic effect, results should be interpreted with caution due to the limited quantitative evidence base, heterogeneity among PBM protocols, and variability in study methodology.

## Figures and Tables

**Table 1:** Characteristics and extracted VAS data of included studies.

Author and Year	Design and Setting	Sample (M/F)	Mean Age (Year)	Laser Type	Application (Intra–Extraoral)	Pain Scale	Time Points	VAS Data (Mean SD)	Main Results
Pérez et al. 2025 [17]	Split-mouth RCT, double blind; Spain	37 (12/25)	22.8 ± 3.6	980 nm diode	Intra + extra at 0–24–48–72 h	VAS 0–10	24/48/72 h	24 h 4 ± 3 vs. 6 ± 3; 48 h 4 ± 3 vs. 5 ± 2; 72 h 2 ± 2 vs. 4 ± 2	Significant reduction in pain at all time points
Camolesi et al. 2025 [12]	Randomized split-mouth; Spain	83 (40/43)	47 (18–65)	808 nm diode	Single postoperative session intra + extra	VAS 0–10	24/48/72 h	24 h 3.56 ± 1.91 vs. 4.85 ± 2.22; 48 h 2.31 ± 1.60 vs. 4.53 ± 2.01; 72 h 1.79 ± 1.51 vs. 3.83 ± 1.98	Significant pain reduction vs. control
Sekerci et al. 2025 [19]	Split-mouth RCT; Switzerland	20 (9/11)	20.4 ± 1.9	670 nm diode	Intra + extra immediate	VAS 0–10	3 d/7 d	N.A.	No significant difference
Uzeda et al. 2025 [6]	Double-blind RCT; Brazil	30	NR	660/808 nm	Pre and immediate	VAS 0–10	7 d	N.A.	No significant difference
Neelofar et al. 2022 [20]	Split-mouth RCT; India	9 (6/3)	25.9 ± 6.7	810 nm diode	Intra + extra 2–4 days	VAS 0–10	2–3–4–7 d	N.A.	Lower pain, non-significant
Momeni et al. 2021 [16]	Double-blind randomized clinical trial	25 (15/10)	24.08 ± 3.26	940 nm diode	Immediate intra	VAS 0–10	1 d/7 d	N.A.	Significant pain reduction at sixth and seventh days
Farhadi et al. 2017 [15]	Double-blind RCT; Iran	48 (24/24)	24.3 ± 2.1	550 nm diode	Immediate intra + extra	VAS 0–10	1 d/7 d	N.A.	No significant effect
Ferrante et al. 2012 [21]	RCT; Italy	30 (15/15)	NR	980 nm diode	Immediate and 24 h	VAS 0–10	1 d/7 d	N.A.	Improved trismus/swelling

N.A.: Not available; VAS: Visual Analog Scale.

**Table 2:** Characteristics of photobiomodulation protocols of the included studies.

Author and Year	Laser Type	Output Power	Energy	Energy Density	Spot Size/Irradiation Area	Irradiation Time	Application Sites	Intraoral/Extraoral Application	Treatment Schedule
Ferrante et al., 2013 [21]	Diode laser (G-Laser 25)	300 mW	54 J	NR	600 μm handpiece	180 s (60 s × 3)	Lingual and vestibular areas; masseter muscle insertion point	Intraoral + extraoral	Immediately after surgery and at 24 h
Farhadi et al., 2017 [15]	Diode laser	NR	2.5 J/area	5 J/cm^2^	0.5 cm^2^	25 s/area	Tooth socket and masseter muscle insertion point	Intraoral + extraoral	Single session immediately after surgery
Momeni et al., 2021 [16]	Diode laser (BIOLASE)	500 mW	NR	10 J/cm^2^ per point (30 J/cm^2^ total treated area)	NR	30 s/point (3 points; total 90 s)	3 intraoral points (occlusal, buccal, and lingual) around extraction site	Intraoral, non-contact technique	Immediately after suturing
Neelofar et al., 2022 [20]	Diode laser (AMD Picasso Lite Dental Diode Laser)	100 mW	NR	4 J/cm^2^	0.3 cm tip (intraoral); 1 × 3 cm handpiece (extraoral)	30 s	Extraction site; masseter muscle origin/insertion and along muscle length	Intraoral + extraoral	Postoperative application
Camolesi et al., 2025 [12]	Ga-Al-As diode laser	100 mW	3 J/point	NR	NR	30 s/point	NR	Intraoral + extraoral	Single postoperative session
Pérez et al., 2025 [17]	Diode laser (PIOON Unilase, Wuhan, China)	100 mW intraoral/lymph node activation; 200 mW extraoral	3 J intraoral/lymph node activation; 8 J extraoral	7.89 J/cm^2^ intraoral/lymph node activation; 1.62 J/cm^2^ extraoral	0.38 cm^2^ intraoral/lymph node; 4.91 cm^2^ extraoral	30 s intraoral/lymph node region; 40 s extraoral	Alveoli; preauricular, submandibular, submental, and cervical lymph node regions; extraoral points	Intraoral + extraoral	Immediately after surgery and repeated at 24, 48 and 72 h
Uzeda et al., 2025 [6]	NR	NR	NR	NR	NR	20 s/point	6 intraoral (MB, DB, ML, DL, MO, DO), 2 extraoral (ant. and post. masseter)	Intraoral + extraoral	Before and immediately after surgery
Sekerci et al., 2025 [19]	GaAlInP diode laser (MED-701)	300 mW	18 J	NR	NR	60 s	Intraoral application and extraoral masseter insertion point	Intraoral + extraoral	Single postoperative session

NR: not reported in the original publication.

**Table 3:** GRADE Summary of Findings.

Time Point	Effect Size (Cohen d, 95% CI)	Certainty of Evidence (GRADE)
24 h	−0.64 (−0.90 to −0.38)	MODERATE
48 h	−0.82 (−1.64 to −0.01)	LOW
72 h	−1.11 (−1.38 to −0.84)	MODERATE

## Data Availability

No new primary data were generated in this study. The data analyzed in the meta-analysis were derived from previously published studies and are available in the original publications cited in the manuscript. No new data were created or analyzed in this study. Data sharing is not applicable to this article.

## Supplementary Materials

The following supporting information can be downloaded at https://www.mdpi.com/article/10.3390/dj14090588/s1, Table S1: Complete search strategies used for the electronic databases; Table S2: PRISMA 2020 Checklist [26].

## Abbreviations

The following abbreviations are used in this manuscript:

PBM	Photobiomodulation
RCT	Randomized controlled trial
VAS	Visual analog scale
SMDs	Standardized mean differences
95%CI	95% confidence interval
ATP	Adenosine triphosphate
PICO	Patient intervention comparison outcome
SD	Standard deviation
RoB	Risk of bias
