# Supplementary material for: The Effect of Photobiomodulation Therapy in the Treatment of Postoperative Pain After Impacted Third Lower Molar Extraction: A Systematic Review with Meta-Analysis
Source: Dent J (Basel). 2026 Sep 11;14(9):588. doi: 10.3390/dj14090588 (PMC13605238; doi:10.3390/dj14090588)
Supplement: Supplementary file 1 [file dentistry-14-00588-s001.zip › Table S1.pdf]

Supplementary Table S1: Complete search strategies used for the electronic databases.

Database	Search strategy
**PubMed**	**Search terms:** third lower molar AND post operative pain AND photobiomodulation. **Filter:** Randomized Controlled Trial. (("third"[All Fields] OR "thirds"[All Fields]) AND ("lower"[All Fields] OR "lowered"[All Fields] OR "lowering"[All Fields] OR "lowerings"[All Fields] OR "lowers"[All Fields]) AND ("molar"[MeSH Terms] OR "molar"[All Fields] OR "molars"[All Fields] OR "molar s"[All Fields]) AND (("postoperative period"[MeSH Terms] OR ("postoperative"[All Fields] AND "period"[All Fields]) OR "postoperative period"[All Fields] OR ("post"[All Fields] AND "operative"[All Fields]) OR "post operative"[All Fields]) AND ("pain"[MeSH Terms] OR "pain"[All Fields])) AND ("low level light therapy"[MeSH Terms] OR ("low level"[All Fields] AND "light"[All Fields] AND "therapy"[All Fields]) OR "low level light therapy"[All Fields] OR "photobiomodulation"[All Fields])) AND (randomizedcontrolledtrial[Filter])
**Embase**	('third lower molar' OR (third AND lower AND ('molar'/exp OR molar))) AND ('post operative pain'/exp OR 'post operative pain' OR (post AND operative AND ('pain'/exp OR pain))) AND ('low level laser therapy'/exp OR 'low level laser therapy') AND 'randomized controlled trial'/de
