# Supplementary material for: The Effect of Photobiomodulation Therapy in the Treatment of Postoperative Pain After Impacted Third Lower Molar Extraction: A Systematic Review with Meta-Analysis
Source: Dent J (Basel). 2026 Sep 11;14(9):588. doi: 10.3390/dj14090588 (PMC13605238; doi:10.3390/dj14090588)
Supplement: Supplementary file 1 [file dentistry-14-00588-s001.zip › Table S2.pdf]

PRISMA 2020 Checklist

Section and Topic	Item #	Checklist item	Location where item is reported
**TITLE**			
Title	1	Identify the report as a systematic review.	p. 1 – Title page (title identifies the report as a systematic review with meta-analysis).
**ABSTRACT**			
Abstract	2	See the PRISMA 2020 for Abstracts checklist.	p. 2 – Abstract (Background/Objectives, Methods, Results, Conclusions).
**INTRODUCTION**			
Rationale	3	Describe the rationale for the review in the context of existing knowledge.	p. 3–4 – Introduction, paragraphs 1–5 (rationale on post-operative pain, PBM mechanism of action and existing evidence gap).
Objectives	4	Provide an explicit statement of the objective(s) or question(s) the review addresses.	p. 4–5 – Introduction, final paragraph ("the present systematic review and meta-analysis was designed to evaluate whether intraoral PBM therapy...").
**METHODS**			
Eligibility criteria	5	Specify the inclusion and exclusion criteria for the review and how studies were grouped for the syntheses.	p. 5–6 – Section 2, "Eligibility criteria (PICO framework)", "Study design", "Inclusion Criteria" and "Exclusion Criteria".
Information sources	6	Specify all databases, registers, websites, organisations, reference lists and other sources searched or consulted to identify studies. Specify the date when each source was last searched or consulted.	p. 6 – Section 2, "Information sources and search strategy" (PubMed and Embase searched up to 18/01/2025).
Search strategy	7	Present the full search strategies for all databases, registers and websites, including any filters and limits used.	p. 6 – Section 2, "Information sources and search strategy" (full PubMed and Embase search strings

Section and Topic	Item #	Checklist item	Location where item is reported
			reported).
Selection process	8	Specify the methods used to decide whether a study met the inclusion criteria of the review, including how many reviewers screened each record and each report retrieved, whether they worked independently, and if applicable, details of automation tools used in the process.	p. 7 – Section 2, "Study selection" (two independent reviewers, LM and LR; disagreements resolved by discussion).
Data collection process	9	Specify the methods used to collect data from reports, including how many reviewers collected data from each report, whether they worked independently, any processes for obtaining or confirming data from study investigators, and if applicable, details of automation tools used in the process.	p. 7 – Section 2, "Data extraction".
Data items	10a	List and define all outcomes for which data were sought. Specify whether all results that were compatible with each outcome domain in each study were sought (e.g. for all measures, time points, analyses), and if not, the methods used to decide which results to collect.	p. 5 and p. 7 – "Eligibility criteria (PICO framework) – Outcome (O)" and Section 2 "Data extraction" (VAS pain values at 24, 48 and 72 h).
	10b	List and define all other variables for which data were sought (e.g. participant and intervention characteristics, funding sources). Describe any assumptions made about any missing or unclear information.	p. 7 – Section 2, "Data extraction" (study characteristics, sample size/demographics, laser parameters and timing).
Study risk of bias assessment	11	Specify the methods used to assess risk of bias in the included studies, including details of the tool(s) used, how many reviewers assessed each study and whether they worked independently, and if applicable, details of automation tools used in the process.	p. 7–8 – Section 2, "Risk of bias assessment" (Cochrane RoB 2 tool, five domains, two independent reviewers).
Effect measures	12	Specify for each outcome the effect measure(s) (e.g. risk ratio, mean difference) used in the synthesis or presentation of results.	p. 7 – Section 2, "Statistical analysis" (Standardized Mean Difference / Cohen's d).
Synthesis methods	13a	Describe the processes used to decide which studies were eligible for each synthesis (e.g. tabulating the study intervention characteristics and comparing against the planned groups for each synthesis (item #5)).	p. 7 – Section 2, "Statistical analysis" (quantitative synthesis restricted to trials reporting mean ± SD for VAS pain).
	13b	Describe any methods required to prepare the data for presentation or synthesis, such as handling of missing summary statistics, or data conversions.	Not explicitly reported – no specific data-conversion or missing-data handling procedure is described.

PRISMA 2020 Checklist

Section and Topic	Item #	Checklist item	Location where item is reported
	13c	Describe any methods used to tabulate or visually display results of individual studies and syntheses.	p. 7 and p. 9 – "Statistical analysis" (forest plots per time point) and Table 1 (tabulated study/VAS data).
	13d	Describe any methods used to synthesize results and provide a rationale for the choice(s). If meta-analysis was performed, describe the model(s), method(s) to identify the presence and extent of statistical heterogeneity, and software package(s) used.	p. 7 – Section 2, "Statistical analysis" (random-effects model implemented in SPSS; heterogeneity via I^2 and Q test).
	13e	Describe any methods used to explore possible causes of heterogeneity among study results (e.g. subgroup analysis, meta-regression).	Not performed – no subgroup analysis or meta-regression was conducted (small number of pooled studies, as acknowledged in the Discussion, p. 16).
	13f	Describe any sensitivity analyses conducted to assess robustness of the synthesized results.	Not performed – no sensitivity analysis is reported.
Reporting bias assessment	14	Describe any methods used to assess risk of bias due to missing results in a synthesis (arising from reporting biases).	Not explicitly described in Methods; addressed only in Results/GRADE section (p. 13: "Publication bias: not assessed due to fewer than 10 studies").
Certainty assessment	15	Describe any methods used to assess certainty (or confidence) in the body of evidence for an outcome.	p. 8 – Section 2, "Certainty of evidence (GRADE)".
**RESULTS**			
Study selection	16a	Describe the results of the search and selection process, from the number of records identified in the search to the number of studies included in the review, ideally using a flow diagram.	p. 8–9 – Section 3, "Study selection" and Figure 1 (PRISMA 2020 flow diagram: 44 records identified, 8 studies included).
	16b	Cite studies that might appear to meet the inclusion criteria, but which were excluded, and explain why they were excluded.	Not reported.
Study characteristics	17	Cite each included study and present its characteristics.	p. 9 – Section 3, "Characteristics of included studies" and

PRISMA 2020 Checklist

Section and Topic	Item #	Checklist item	Location where item is reported
			Table 1.
Risk of bias in studies	18	Present assessments of risk of bias for each included study.	p. 9–10 – Section 3, "Risk of bias", Figure 2 (summary graph) and Figure 3 (traffic-light plot, RoB 2).
Results of individual studies	19	For all outcomes, present, for each study: (a) summary statistics for each group (where appropriate) and (b) an effect estimate and its precision (e.g. confidence/credible interval), ideally using structured tables or plots.	p. 9 – Table 1 (VAS mean ± SD by study); individual effect estimates presented only for the two studies pooled in the meta-analysis (Figures 4–6).
Results of syntheses	20a	For each synthesis, briefly summarise the characteristics and risk of bias among contributing studies.	p. 12 – Section 3, "Quantitative synthesis" (introductory paragraph).
	20b	Present results of all statistical syntheses conducted. If meta-analysis was done, present for each the summary estimate and its precision (e.g. confidence/credible interval) and measures of statistical heterogeneity. If comparing groups, describe the direction of the effect.	p. 12 – Section 3, "Pain at 24 hours", "Pain at 48 hours" and "Pain at 72 hours", with Figures 4–6 (forest plots).
	20c	Present results of all investigations of possible causes of heterogeneity among study results.	Not applicable
	20d	Present results of all sensitivity analyses conducted to assess the robustness of the synthesized results.	Not applicable
Reporting biases	21	Present assessments of risk of bias due to missing results (arising from reporting biases) for each synthesis assessed.	p. 13 – Section 3, "Certainty of evidence" (publication bias not assessed owing to the small number of studies).
Certainty of evidence	22	Present assessments of certainty (or confidence) in the body of evidence for each outcome assessed.	p. 13 – Section 3, "Certainty of evidence" and Table 2 (GRADE Summary of Findings).
**DISCUSSION**			
Discussion	23a	Provide a general interpretation of the results in the context of other evidence.	p. 14 – Section 4, Discussion, opening paragraphs (interpretation of the pooled analgesic effect of PBM).
	23b	Discuss any limitations of the evidence included in the review.	p. 14–16 – Section 4, Discussion

PRISMA 2020 Checklist

Section and Topic	Item #	Checklist item	Location where item is reported
			(heterogeneity of PBM protocols, non-parametric/non-extractable outcome reporting, RoB concerns).
	23c	Discuss any limitations of the review processes used.	p. 16 – Section 4, Discussion ("Several limitations of this review must be acknowledged...", small number of quantitative studies, publication bias not assessable).
	23d	Discuss implications of the results for practice, policy, and future research.	p. 16 – Section 4, Discussion (clinical relevance paragraph) and Section 5, Conclusions.
**OTHER INFORMATION**			
Registration and protocol	24a	Provide registration information for the review, including register name and registration number, or state that the review was not registered.	p. 5 – Section 2, "Protocol and registration" (PROSPERO registration number CRD420261285303).
	24b	Indicate where the review protocol can be accessed, or state that a protocol was not prepared.	p. 4
	24c	Describe and explain any amendments to information provided at registration or in the protocol.	Not reported – no amendments to the registered protocol are described.
Support	25	Describe sources of financial or non-financial support for the review, and the role of the funders or sponsors in the review.	p. 17 – "Funding" statement ("This research received no external funding").
Competing interests	26	Declare any competing interests of review authors.	p. 17 – "Conflicts of Interest" statement ("The authors declare no conflicts of interest").
Availability of data, code and other materials	27	Report which of the following are publicly available and where they can be found: template data collection forms; data extracted from included studies; data used for all analyses; analytic code; any other materials used in the review.	p. 17 – "Data Availability Statement" ("No new primary data were generated in this study. The data analyzed in

PRISMA 2020 Checklist

Section and Topic	Item #	Checklist item	Location where item is reported
			the meta-analysis were derived from previously published studies and are available in the original publications cited in the manuscript. Data sharing is not applicable to this article"); analytic code and other materials are not reported as separately available.

From: Page MJ, McKenzie JE, Bossuyt PM, Boutron I, Hoffmann TC, Mulrow CD, et al. The PRISMA 2020 statement: an updated guideline for reporting systematic reviews. BMJ 2021;372:n71. doi: 10.1136/bmj.n71. This work is licensed under CC BY 4.0. To view a copy of this license, visit https://creativecommons.org/licenses/by/4.0/
